# Variation in Meiotic Recombination Frequencies Between Allelic Transgenes Inserted at Different Sites in the *Drosophila melanogaster* Genome

**DOI:** 10.1534/g3.113.006411

**Published:** 2013-08-01

**Authors:** Susan McMahan, Kathryn P. Kohl, Jeff Sekelsky

**Affiliations:** * Department of Biology, University of North Carolina at Chapel Hill, Chapel Hill, North Carolina 27599; § Curriculum in Genetics and Molecular Biology, University of North Carolina at Chapel Hill, Chapel Hill, North Carolina 27599; † Program in Molecular Biology and Biotechnology, University of North Carolina at Chapel Hill, Chapel Hill, North Carolina 27599

**Keywords:** meiotic recombination, *Drosophila*

## Abstract

Meiotic crossovers are distributed nonrandomly across the genome. Classic studies in *Drosophila* suggest that the position of a gene along a chromosome arm can affect the outcome of the recombination process, with proximity to the centromere being associated with lower crossing over. To examine this phenomenon molecularly, we developed an assay that measures meiotic crossovers and noncrossover gene conversions between allelic transgenes inserted into different genomic positions. To facilitate collecting a large number of virgin females, we developed a useful genetic system that kills males and undesired classes of females. We found that the recombination frequency at a site in the middle of the *X* chromosome, where crossovers are normally frequent, was similar to the frequency at the centromere-proximal end of the euchromatin, where crossovers are normally infrequent. In contrast, we recovered no recombinants—crossovers or noncrossovers—at a site on chromosome *4* and at a site toward the distal end of the *X* chromosome. These results suggest that local sequence or chromatin features have a stronger impact on recombination rates in this transgene assay than position along the chromosome arm.

Meiotic recombination serves several important functions. First, in many organisms the pairing of homologous chromosomes in meiosis is dependent on recombination (reviewed in Bhalla and Dernburg 2008). Second, chiasmata resulting from crossovers promote accurate segregation of homologous chromosomes at the reductional division, allowing the production of gametes or spores with the correct haploid number of chromosomes (reviewed in Martinez-Perez and Colaiácovo 2009). Third, recombination produces new combinations of alleles at different loci, resulting in greater genetic variation upon which natural selection can act and allowing detrimental mutations to be removed from chromosomes bearing favorable alleles at other loci (reviewed in Otto and Lenormand 2002).

Not all meiotic recombination events are equally able to achieve each of these benefits. For example, crossovers promote disjunction of homologous chromosomes, but noncrossover recombination events do not. Moreover, some crossovers may be more capable of promoting disjunction than others. Studies of meiotic nondisjunction of human chromosome *21* and the *Drosophila X* chromosome suggested that a single distal crossover is less able to ensure correct disjunction than a single medial crossover and that extremely proximal crossovers actually disrupt proper segregation (Koehler *et al.* 1996; Lamb *et al.* 1996). These observations may explain why crossovers tend be more frequent in the medial regions of chromosomes in *Drosophila* and human female meiosis.

Sturtevant (1913) generated the first genetic maps, based on crossover frequencies between pairs of genes, 100 years ago. A decade later, Muller’s (1927) discovery that ionizing radiation induces chromosome rearrangements made it possible to build physical maps of chromosomes. Within a few years several researchers, including Painter and Muller (1929), Dobzhansky (1930), and Beadle (1932), concluded from comparison of physical and genetic maps that the frequency of crossovers per unit distance varies along *Drosophila* chromosomes, with the most noticeable effect being suppression of crossing over near the centromere. Conclusive evidence that this effect is caused by proximity to the centromere was demonstrated by Sturtevant and Beadle (1936), who found that crossover frequencies in the proximal euchromatin increase when this region is moved away from the centromere by inversion. Green (1955) took advantage of this phenomenon to study crossovers between different alleles of *forked* (*f*). This gene is located about a quarter of the distance from the pericentric heterochromatin of the *X* to the telomere, a region where crossovers are relatively infrequent. Green recovered a single crossover among 45,000 progeny of *f^1^* / *f^3n^* females. He then crossed each allele onto *In(1)sc*, which inverts most of the chromosome arm, starting a few hundred kilobases from the telomere and ending in the middle of the pericentric heterochromatin. This moved *forked* much farther from the centromere but left flanking sequences unchanged for at least five megabase pairs in each direction. In this configuration, the frequency of crossovers between *f^1^* and *f^3n^* was elevated eightfold. This result suggests that meiotic crossover frequency is not an intrinsic property of a given gene or region but rather is determined at least in part by the position of that gene or region along a chromosome.

Although different patterns of crossover distribution along chromosomes may be exhibited by different species, or even between the sexes of one species, these patterns are nonetheless highly regulated. The mechanisms used to control crossover distribution are poorly understood (reviewed in Phadnis *et al.* 2011; Youds and Boulton 2011). Meiotic recombination is initiated through the introduction of double-strand breaks (DSBs), so a simple method to control crossover distribution might be to make DSBs exclusively at sites at which crossovers are desired and then generate a crossover at each DSB site. However, there are more DSBs than crossovers, by an order of magnitude or more in some species (*e.g.*, Moens *et al.* 2002). Most meiotic DSBs are repaired through pathways that do not produce crossovers; these noncrossover outcomes can be detected when they involve gene conversion, the unidirectional transfer of information from one DNA duplex to another (reviewed in Andersen and Sekelsky 2010). Thus, to understand crossover control, it is necessary to understand the factors that influence initiation as well as the factors that control the crossover/noncrossover decision.

Studies in *Drosophila* suggest that chromosomal position effects on crossover frequency may be mediated through the crossover/noncrossover decision. Chovnick *et al.* (1993), Smith *et al.* (1970), and Finnerty *et al.* (1970) conducted experiments to dissect the fine-structure of the *maroon-like* (*mal*) gene, which is located toward the centromere-proximal end of the euchromatin, approximately 2 Mb from the pericentric heterochromatin. When *mal* was in its endogenous location, almost all recombination events recovered were noncrossover gene conversions (Smith *et al.* 1970). These researchers then used the aforementioned method used by Sturtevant and Green, crossing *mal* mutations onto inverted chromosomes. In this configuration, intragenic crossovers were recovered much more frequently, even though sequences surrounding *mal* were unchanged for several megabases to the right (including a large block of pericentric heterochromatin) and about 20 Mb to the left (almost the entire euchromatic sequence of the *X* chromosome) (Finnerty *et al.* 1970). This finding suggests that the longitudinal position of a gene along a chromosome arm affects either the total frequency of recombination (*i.e.*, the frequency of DSBs), the probability that an initiated event becomes crossover, or both. The data do not distinguish between these possibilities because different alleles of *mal* were used in the different experiments.

More recently, fine-scale mapping of crossovers in *Drosophila* have found substantial variation in crossover rates at smaller scales (~5−50 kb) (Cirulli *et al.* 2007; Singh *et al.* 2009; Comeron *et al.* 2012; Singh *et al.* 2013). Several genomic features, including sequence motifs and gene content, were found to be significantly associated with greater crossover rates (Comeron *et al.* 2012; Miller *et al.* 2012; Singh *et al.* 2013). In contrast, the frequency of noncrossover gene conversion appears to be similar throughout the euchromatic genome (Comeron *et al.* 2012), which suggests that the distribution of DSBs is much more uniform than the distribution of crossovers. Considered in light of the aforementioned genetic studies, these findings suggest that chromosomal position has a major influence on the crossover/noncrossover decision.

We sought to develop a system that would allow us to use genetic and molecular tools to understand the effects of chromosomal position on the crossover/noncrossover decision. We chose *rosy* (*ry*) because it is possible to select for rare wild-type recombination products from females heteroallelic for *ry* mutations, allowing detailed studies of intragenic recombination (Chovnick *et al.* 1993). The endogenous *ry* gene is in the middle of chromosome arm *3R*. We made use of technology that allows integration of different transgenes into the same genomic location to assay recombination within *ry* at four different genomic locations: proximal, medial, and distal sites on the *X* chromosome, and a site on chromosome *4*. Surprisingly, recombination rates were identical at the proximal and medial *X* chromosome locations, and recombination was completely absent at the distal *X* and chromosome *4* sites. These findings suggest that short-range effects on recombination are stronger than effects of position along the chromosome in this assay.

## Materials and Methods

### Generation of transgenes

Descriptions of *ry* follow the coordinate system of Coté *et al.* (1986), in which +1 is within an *Eco*RI site in the second exon. This corresponds to 3R:8,859,890 on the genome assembly (release 5.44). Sequences toward the 3′ end of *ry* are positive coordinates and are also increasing coordinates on the genome assembly.

To generate the *ry+* transgene, the following fragments were cloned into the pMTL23P cloning vector (Chambers *et al.* 1988): (1) a 285-bp phiC31 *attB* sequence; (2) the *3xP3*::*dsRed* marker gene from pM{3xP3-RFPattP} (Bischof *et al.* 2007), including the downstream *loxP* site; and (3) the 7289 bp *ry* genomic *Hin*dIII rescue fragment (*ry^+t7.2^*) from pP{Car20} (Rubin and Spradling 1982), spanning −3131 to +4158. The product, pRatt-ry, is 12,287 bp. To generate pRatt-ry606N, the segment from a *Pme*I site at −759 to an *Sfi*I site at +975 was replaced with the same fragment amplified from a *ry^606^* mutant fly. This region carries the *ry^606^* mutation (G to A at −468). In addition, since the *ry^+t7.2^* fragment came from a *ry^+5^* fly and the *ry^606^* mutation was induced on a *ry^+6^* chromosome, this exchange results in several polymorphisms between pRatt-ry606N and pRatt-ry609N (Table 1). To generate pRatt-ry609N, the *ry^609^* mutation (G to A at 3506) was introduced by site-directed mutation. At the same time, A at 3511 was changed to T, a silent mutation that generates a *Bgl*II site overlapping *ry^609^*. Additional polymorphisms were generated by site-directed mutagenesis (Table 1).

**Table 1:** Polymorphisms in pRatt-ry transgenes

Position*^a^*	*ry+*	*ry^606N^*	*ry^609N^*	Comments
−3149	GTAC	−	GTAC	Destroys *Kpn*I site for flanking marker
−2914	G	G	C	Destroys *Pst*I site
−710	C	A	C	From *ry^606^* chromosome
−679	A	G	A	From *ry^606^* chromosome
−527	G	G	T	From *ry^606^* chromosome
−468	G	G	A	*ry^606^* mutation
−332	T	T	G	From *ry^606^* chromosome
−323	T	T	C	From *ry^606^* chromosome
−320	A	A	C	From *ry^606^* chromosome
+73	G	T	G	From *ry^606^* chromosome
+239	C	T	C	From *ry^606^* chromosome
+355	T	A	T	From *ry^606^* chromosome
+449	T	C	T	From *ry^606^* chromosome
+467	C	T	C	From *ry^606^* chromosome, creates *Age*I site
+1103	C	C	G	Destroys *Pst*I site (with +1103)
+1106	C	C	T	Destroys *Pst*I site (with +1106)
+2950	A	A	G	Destroys *Bam*HI site
+3358	C	C	G	Destroys *Sph*I site
+3506	G	G	A	*ry^609^* mutation
+3511	A	A	T	Creates *Bgl*II site (with +3506)
+3610	C	C	A	Creates *Eco*RI site
+3735	A	A	C	Creates *Bam*HI site
+4163	−	−	GATATCGAATT	Inserts *Eco*RV and *Eco*RI sites
+4169	C	C	G	Destroys *Nhe*I site
+6998	C	C	G	Destroys *Age*I site for flanking marker

a Positions are according to Coté *et al.* (1986); +1 is 3R:8,859,890 on genome assembly release 5.44. Coordinates from −3131 to +4158 are genomic DNA from the *ry* region. Sequences outside of this region are from the vector.

To generate pWhiteReaper, a 235-bp sequence containing the *rpr* coding region was amplified from genomic DNA and cloned into pUAST (Rørth *et al.* 1998), generating pUAST-rpr. The following fragments were then cloned into the pMTL23P cloning vector (Chambers *et al.* 1988): (1) a fragment from pStinger (Barolo *et al.* 2000) containing *eGFP-nls* flanked by *gypsy* insulator sequences; (2) a 985-bp fragment containing the *tub84B* promoter, first exon, and first intron, inserted upstream of *eGFP-nls*; (3) a phiC31 *attB* site, inserted outside of the upstream insulator; (4) a 1593-bp fragment from pUAST-rpr, containing the UAS enhancers, *hsp70* promoter, *rpr* coding sequence, and SV40 3′ UTR, between the upstream insulator and the *tub*::*GFP* gene. The resulting plasmid, pGreenReaper, is 7624 bp. We were unsuccessful in obtaining integrations of this plasmid into the *Y* chromosome *P*{*Cary.attP*} landing site after screening for green fluorescence. We then generated pWhiteReaper by replacing most of the *tub*::*GFP* gene with a fragment in which the *hsp70* basal promoter was fused to a *w* cDNA. Euchromatic transformants with this *w^+70c^* chimeric gene have a nearly wild-type eye color regardless of genomic insertion location (S. McMahan and J. Sekelsky, unpublished data).

### Tests of purine treatment for transgenes

To determine whether flies carrying a *ry+* transgene are able to survive purine treatment, we set up bottles with 30 *y*; *kar ry^506^ cv-c* virgin females crossed to 10 males whose genotype was wild-type (endogenous *ry*), hemizygous for *M*{*ry^+^*}*(*2A, 6E, or 20C), and homozygous for *ry^506^* or homozygous for *M*{*ry^+^*}(102D) and for *ry^506^*. For each genotype, three bottles were treated with purine as described in the section *Intragenic recombination assays* and three were untreated. Adult males and females that emerged were counted. Two bottles (one treated, one untreated) for 102D were discarded because of contamination with an unknown pathogen that causes larval lethality. Treated and untreated counts were compared in an unpaired *t*-test with the use of Prism 6.02 (Graphpad), with each bottle considered a biological replicate. For wild-type and 102D, male and female counts were summed before doing the *t*-test, but for the *X* insertions males and females were compared separately because males did not inherit a transgene and therefore did not survive the treatment with purine.

### Intragenic recombination assays

Each transgene was inserted into each of four landing sites: *M*{*3xP3-RFP.attP*’}*ZH-6E*, *M*{*3xP3-RFP.attP*’}*ZH-20C*, *M*{*3xP3-RFP.attP*’}*ZH-102D*, and *M*{*3xP3-RFP.attP*’}*ZH-2A*. Before integration, we removed the *3xP3*::*RFP* gene from the landing site by Cre-mediated recombination between flanking *loxP* sites. This allowed us to use the *3xP3*::*RFP* gene on our construct as a marker for transformation. For sites on the *X* chromosome, we built stocks homozygous for the landing site construct and the *M*{*vas-int.Dm*}*ZH-102D* integrase construct on chromosome *4*. For the site on chromosome *4*, the stock was homozygous for this site and for the *M*{*vas-int.Dm*}*ZH-2A* integrase construct. After injection, the integrase was crossed out and stocks were made homozygous for *ry^506^* on chromosome *3*. In stocks with the *ry^606N^* transgene, this chromosome also carried *P*{*GawB*}*h^1J3^*. In stocks with the *ry^609N^* transgene, the *Y* chromosome carried *P*{*UAS-rpr.Y*}.

Virgin females heteroallelic for *ry^606N^* and *ry^609N^* constructs were crossed to *y/y+Y* ; *kar ry^506^ cv-c* males. Crosses were set up in bottles containing 25 mL of food medium using 30 females and 10 males. After 3 d, the adults were transferred to new bottles to generate the second brood, and 0.75 mL of 0.2% purine was added to the first brood bottles. Three days later adults were removed from second brood bottles and discarded, and purine was added to these bottles. In each tray of 25 bottles, one bottle, selected at random, was left untreated. Progeny from this bottle were counted to estimate the total number of progeny screened.

To distinguish crossovers from noncrossovers the flanking *Kpn*I and *Age*I sites were checked by polymerase chain reaction (PCR)-amplifying fragments spanning these sites and digesting with the appropriate enzyme. To map gene conversion tracts in noncrossover recombinants we sequenced PCR fragments spanning the mutant sites (*ry^606N^* or *ry^609N^*).

An online calculator at http://www.graphpad.com/quickcalcs/ was used to determine two-sided *P* values using the Fisher exact test, based on number of recombination events per 1000 progeny (*e.g.*, 112 crossovers from 3710 thousand progeny at the endogenous *ry* location).

## Results and Discussion

### A transgenic system to measure recombination rates between the same allelic pair at different genomic locations

**Figure 1:**
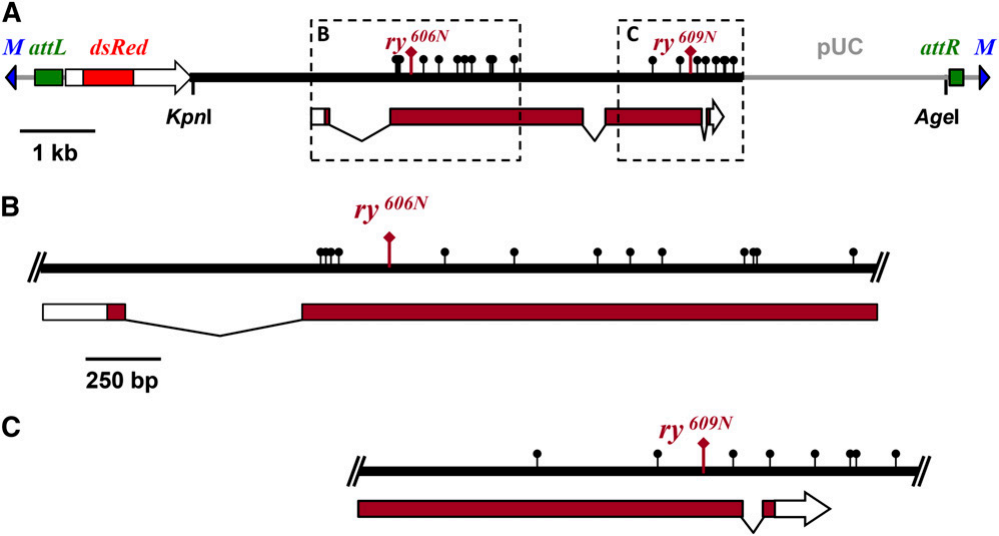
Structure of *ry* transgenes used in this study. (A) Transgenes carried a 7.3-kb genomic fragment spanning the *ry* gene (black line) and *3xP3*::*dsRed* marker for transgenesis. The structure of the *ry* transcript is shown below. Transgenes were integrated into landing sites with *Mariner* (*M*) ends. Integration includes the pUC plasmid backbone. The locations of the *ry^606N^* and *ry^609N^* mutations are indicated, as are the *Kpn*I and *Age*I sites used as flanking markers and polymorphisms introduced for conversion tract mapping (black lollipops). Regions in dotted boxes are magnified in (B) and (C) to show polymorphism distribution (see Table 1 for exact sequence changes).

To assess the effects of genomic position on recombination rates within identical sequences, we used the ϕC31 site-specific recombination system (Bischof *et al.* 2007). This allowed us to insert similar transgenes into allelic positions on homologous chromosomes. We first built a transgene that carries a 7.3-kb wild-type *ry* genomic fragment, a *3xP3*::*RFP* marker gene, and a ϕC31 *attB* site (Figure 1). We used site-directed mutagenesis to generate derivatives with the mutations in *ry^606^* and *ry^609^*. Although *ry^606^* and *ry^609^* are considered to be genetically null when homozygous flies are compared with hemizygous flies, these alleles complement one another for eye color (Gelbart *et al.* 1976). The *ry* gene encodes xanthine dehydrogenase (XDH). Gelbart *et al.* (1976) found XDH activity in *ry^606^*/*ry^609^* mutants to be 2.5% of wild-type levels, likely the result of weak activity of hybrid multimers, since XDH functions as a homodimer and these mutations alter residues in different domains of the polypeptide (Gelbart *et al.* 1976; Hille 1996). Additional sites were modified to provide polymorphisms for mapping gene conversion tracts (Table 1); we refer to these derivative alleles as *ry^606N^* and *ry^609N^*.

To generate flanking markers for distinguishing crossovers from noncrossover gene conversions, we destroyed a *Kpn*I restriction site 2.7 kb upstream of the *ry^606N^* mutation and an *Age*I site 3.5 kb downstream of the *ry^609N^* mutation. In our previous studies of gene conversion at the endogenous *ry* locus, the average span of co-converted markers was 551 ± 98 bp (n = 29), with the longest tract being 2298 bp (Blanton *et al.* 2005). If gene conversion tracts are similar in length in different locations of the genome, the sites we modified will rarely be co-converted with mutant sites, allowing us to use the modified sites as flanking markers to distinguish crossovers from noncrossovers.

Each of the three constructs (*ry^+^*, *ry^606N^*, and *ry^609N^*) was introduced first into a site in cytological region 6E on the polytene chromosome map. Crossovers are relatively frequent in this region of the chromosome, at least on a broad scale. We refer to these insertions as *M*{*ry^+^*}(6E), *M*{*ry^606N^*}(6E), and *M*{*ry^609N^*}(6E). We generated stocks homozygous for each of these transgenes on the *X* chromosome and for *ry^506^*, a deletion that removes most of the endogenous *ry* locus, on chromosome *3*.

### A genetic system to generate virgin females

Because intragenic recombination between *ry* alleles is rare, we screen approximately 10^6^ progeny of *ry* mutant females in a typical experiment (Blanton *et al.* 2005; Radford *et al.* 2007a,b). Collection of the large number of virgin females required to generate these progeny can be labor-intensive. To accelerate this process we developed a system that automatically kills male progeny. An existing method makes use of a *Y* chromosome transgene that expresses the cell death gene *hid* under the control of the *hsp70* (cited in Starz-Gaiano *et al.* 2001). This method is not suitable for our purposes because heat shock affects recombination frequencies (Plough 1917) and because the effectiveness (percentage of males killed) is variable in our laboratory. As an alternative, we generated a *Y* chromosome carrying a *rpr* cell death transgene under the control of a Gal4-inducible promoter, similar to the *P*{*w^+mC^* = UAS-rpr.C} transgene of Aplin and Kaufman (1997). It can be difficult to recover transpositions of *P* elements onto the *Y* chromosome, so we rebuilt a *UAS*::*rpr* fusion gene and cloned it into vectors for φC31-mediated transgenesis (see *Materials and Methods*). This was then integrated into a pre-existing landing site on the *Y* chromosome (S. Russell, personal communication), producing *Y*, *P*{*w^+c^* = UAS-rpr.Y}.

**Figure 2:**
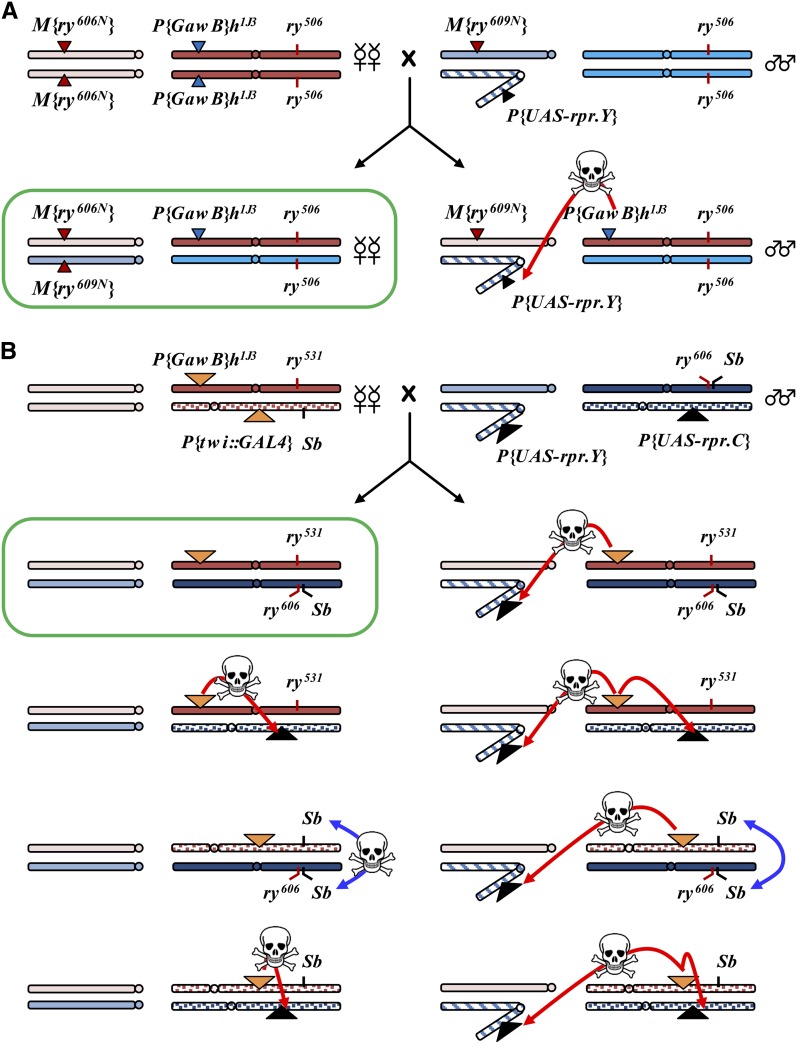
Self-virgining cross schemes. (A) Scheme to generate *M*{*ry^606N^*} / *M*{*ry^609N^*} virgins. Female chromosomes are red; male chromosomes are blue. Circles indicate centromeres. The *Y* chromosome is hatched, the telocentric chromosome is the *X*, and the metacentric chromosome is *3*. Triangles denote transgene insertions (the position along the chromosome is not meant to indicate the actual insertion location, which is unknown for some insertions). Genotypes of the parents are represented above, and genotypes of the progeny below. The red arrowhead indicates that Gal4 activates expression of *rpr*, resulting in death of all sons; only virgin daughters (circled in green) survive. (B) A more complex scheme to generate virgins with a desired heteroallelic combination of third chromosomes. In this cross, eight classes of progeny are produced, four of each sex. Six of these are killed by Gal4-mediated activation of *rpr*, and seventh dies because of homozygosity for *Sb* (double-headed blue arrow), leaving only the desired virgin females (circled in green).

Males of the genotype *w^1118^* / *Y*, *P*{*w^+c^* = UAS-rpr.Y} have variegated eyes, indicating that the transgene is subject to position-effect variegation, despite the presence of *gypsy* chromatin insulators on the construct. However, when these males are crossed to *P*{*GawB*}*h^1J3^* / *TM3*, *Sb* females, no males inheriting the *P*{*GawB*}*h^1J3^* chromosome survive to adulthood: The combination of *P*{*GawB*}*h^1J3^*, which expresses Gal4 in the pattern of the *hairy* (*h*) gene, and *Y*, *P*{*w^+c^* = UAS-rpr.Y} is completely lethal. We used the crossing scheme shown in Figure 2A to take advantage of this lethality and generate virgin females for our recombination experiments.

To extend the functionality of this system, we jumped an existing *X* chromosome insertion of *P*{*w^+mC^* = UAS-rpr.C} onto chromosome *2* and *3* balancers. Both insertions cause complete lethality when combined with *P*{*GawB*}*h^1J3^*. The *UAS*::*rpr* transgenes are also lethal when combined with *TM3*, *P*{*w^+mC^* = GAL4-twi.G}2.3, *P*{UAS-2xEGFP}AH2.3, *Sb Ser*. This facilitates more complex crosses like the one shown in Figure 2B. We used this scheme in another recombination assay and were able to generate 145,000 virgin females with relative ease (K. P. Kohl and J. Sekelsky, unpublished data). In some crosses there are surviving males carrying *Y*, *P*{*w^+c^* = UAS-rpr.Y} and a Gal4 driver, but these are still infrequent. Stocks with the chromosomes described above have been deposited into the Bloomington *Drosophila* Stock Center.

### Recombination within *ry* transgenes at ectopic locations

**Figure 3:**
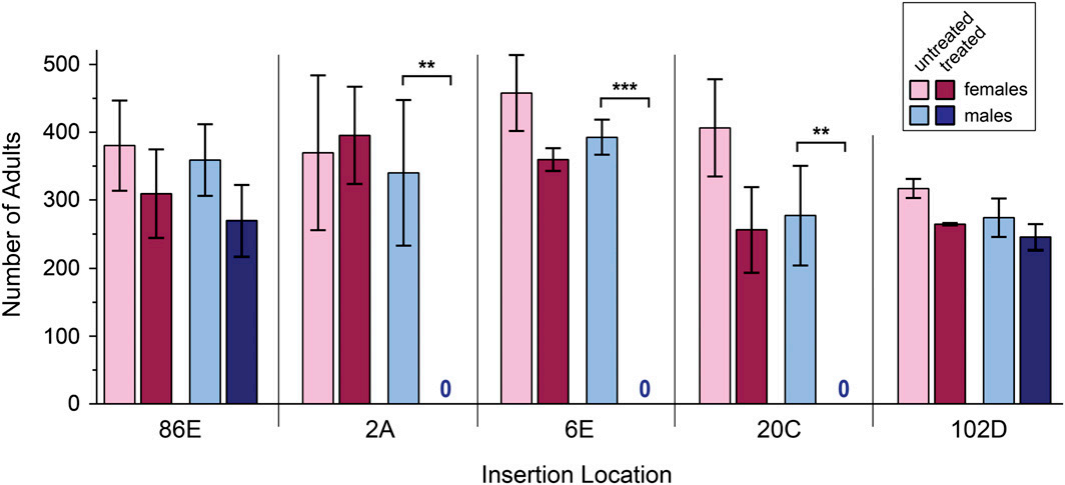
Effects of purine selection on recovery of adult flies. For each location, six bottles were set up with *y*; *kar ry^506^ cv-c* females, which were crossed to males hemizygous (*X* insertions) or homozygous (102D) for a transgene, or to wild-type males (endogenous site). Three bottles were treated with purine and three were left untreated. Bars show the mean number of females and males recovered from each. The only significant differences were for males for *X* insertions. In these cases only daughter received the transgene, so no males were recovered from treated bottles. Error bars are standard deviation. n = 3 in all cases except for 102D, where n = 2 because two bottles were contaminated and were excluded. **P < 0.01. ***P < 0.0001. All other pairwise comparisons were considered not statistically significant (*P* > 0.05).

Before assaying recombination, we asked whether local position effects alter functionality of the *ry* transgenes. Because selection demands expression of a functional *ry* gene, we confirmed that *M*{*ry^+^*}6E; *ry^506^* larvae survive purine selection. Flies that are heterozygous for the *ry^506^* deletion are completely viable after purine treatment (Figure 3). Similarly, flies homozygous for *ry^506^* but carrying one copy of a *ry+* transgene, which is the genotype that is selected for in our experiments, are completely viable at 6E and at each of the other insertion sites tested. Additional support for complete function of the transgenes comes from observations of heteroallelic genotypes. Flies heteroallelic for *ry^606^* and *ry^609^* have 2.5% of the wild-type level of XDH activity, which is sufficient for wild-type eye color but insufficient for survival on purine selection (Gelbart *et al.* 1976). The alleles we constructed *in vitro* recapitulate this behavior when inserted into the 6E site as well as other sites used in this study. Thus, the different genomic locations we used do not cause any observable changes in *ry* function compared to the endogenous location.

We used a purine selection experiment to measure the frequency of intragenic meiotic recombination. In previous studies of the endogenous *ry* locus in this laboratory, 112 crossovers and 53 noncrossover gene conversions were recovered after screening 3.7 × 10^6^ progeny (Blanton *et al.* 2005; Radford *et al.* 2007a,b). At the 6E site, we recovered six crossovers and ten gene conversions in a screen of 943,000 progeny (Table 2). Although the frequency of noncrossover gene conversion was not significantly different between these experiments (*P* = 0.53), crossovers were about fivefold more frequent at the endogenous locus than at 6E (*P* < 0.0001), despite the fact that both locations are in medial positions along the chromosome arm (Table 2).

**Table 2:** Intragenic recombination rates at different genomic locations

Location*^a^*	Progeny Screened	Crossovers		Noncrossovers		
		*n*	Rate	*n*	Rate	Tract Length*^c^*
86E (3R:8859889)*^b^*	3,710,000	112	3.0 × 10^−5^	53	1.4 × 10^−5^	800 ± 117
6E (X:6845474)	943,000	6	6.3 × 10^−6^	10	1.1 × 10^−5^	987 ± 260
20C (X:22221488)	903,500	10	1.1 × 10^−5^	7	7.7 × 10^−6^	889 ± 257
2A (X:1346142)	960,600	0	–	0	–	–
102D (4:1008975)	1,285,800	0	–	0	–	–

a Coordinates are from genome assembly release 5.44.

b This is the endogenous *ry* location (coordinate is the first base pair of the *Eco*RI site). Data are from Blanton *et al.* (2005) and Radford *et al.* (2007b).

c Tract lengths, in base pairs, were calculated as the midpoint between polymorphic sites that were converted and the nearest polymorphic sites that were not converted. Numbers in the table are mean ± SEM.

Several factors may contribute to the differences between crossover rates at the endogenous *ry* locus and between our transgenes at 6E. These include chromosomal location, chromatin environment, strain differences (background effects), and local sequence context, including the composition of the transgene or the *Mariner* landing site. To determine whether chromosome position affects recombination rates, we repeated the experiment with transgenes located in 20C, only 200 kb from the end of the assembled euchromatic sequence. Because crossovers are less frequent in proximal regions of the *X* than in medial regions, we hypothesized that the *ry* transgenes integrated into 20C would have a lower crossover rate than at 6E. We screened a similar number of progeny of *M*{*ry^609N^*} / *M*{*ry^606N^*} females at the 20C site and, surprisingly, recovered a similar number of recombinants (Table 2). At 20C, as at 6E, crossovers were reduced relative to the endogenous *ry* locus (*P* = 0.0010), but noncrossover gene conversions were not (*P* = 0.14). There was no significant difference between the frequencies of recombinants at 6E and 20C.

These results suggest that if there is an effect of chromosomal position (*e.g.*, medial *vs.* proximal) it is small compared with other effects experienced by our transgenes. One such effect might arise from sequences on the transgenes themselves: The transgenic *ry* constructs may have properties that are sufficient to dictate recombination frequency regardless of insertion position. To test this hypothesis we measured recombination frequencies at two additional regions in which crossovers are normally low (region 2A toward the distal end of the *X* chromosome) or absent (region 102D on chromosome *4*). We screened 960,000 progeny for the 2A site and 1,285,000 progeny for the 102D site. To our surprise, we recovered no *ry^+^* recombinants – neither crossovers nor noncrossover gene conversions – in either experiment. As at the 6E and 20C sites, *ry* function appeared to be normal at these locations: The *ry^+^* transgene conferred survival through purine selection (Figure 3) and *M*{*ry^609N^*} and *M*{*ry^606N^*} complemented for eye color but not for survival after purine treatment. Insertion locations were verified by PCR with primers in the flanking genomic sequence and in the transgene. For the 2A site, we also crossed the *y* mutation off of the *M*{*ry^609N^*} and crossed *ec* onto the *M*{*ry^606N^*} chromosome. The ability to obtain the desired recombinants suggests that there are no large chromosome rearrangements associated with these transgenes.

To better understand our results, we compared the crossover rate we measured to the average rate in the genomic neighborhood of each transgene (Figure 3). According to the standard map (Lindsley and Zimm 1992), the 1.75-Mb interval from *kar* to *cv-c*, which spans the endogenous *ry* location, is 2.4 map units, for an average of 1.37 map units per Mb. This is not greatly different from the value of 1.60 map units per Mb between *ry^531^* and *ry^606^* (112 crossovers from 3.7 × 10^6^ progeny; only half of the crossovers – those that are *ry+* – are recovered by purine selection). There is also good agreement at the 20C site. We measured a rate of 0.56 map units per Mb in our *ry* transgene experiments at this site. The 2.2-Mb interval from *mal* to *su(f)* is 1.1 map units, which is 0.50 map units per Mb. This interval also includes an unknown length of heterochromatin that has not been assembled, so the actual rate would be lower if we included heterochromatic sequences. It should also be noted that the fine-structure mapping of Comeron *et al.* (2012) shows substantial fine-structure variation in crossover rates in this region. Because regions of high and low crossing over varied between different strains, it is not possible to predict local recombination rates in our experiments. It was also not possible to measure these rates directly because of the absence of polymorphisms between the chromosomes we used due to both being inserted into the same parental landing site chromosome.

In contrast to the endogenous and 20C sites, the 6E site showed a large disparity between the average crossover rate and the rate between our *ry* transgenes. A 1.6-Mb region that spans this insertion site (*rux* to *cm*) is five map units, for an average of 3.13 map units per Mb. In our experiments we measured 0.32 map units per Mb, about a tenth of the average rate in the surrounding neighborhood. At the 2A site, we recovered no crossovers, even though the average rate in this region is about the same as in 20C.

### Factors that influence recombination rates and outcomes

Recombination rates in our assay area appear to be determined primarily by local effects of insertion location, perhaps in combination with properties inherent to the transgenes. There were no significant differences between the 6E and 20C sites for crossover frequency, noncrossover frequency, or conversion tract length (Table 2 and Figure 5). Compared with the endogenous *ry* gene, however, the crossover frequencies at these sites were reduced by approximately 80%, whereas noncrossover frequencies and conversion tract lengths were similar. One possible explanation is that properties of the transgenes themselves set an upper limit on the crossover rate, and that permissive properties of the 6E and 20C insertion sites allow the transgenes to achieve this rate, whereas restrictive properties of the 2A and 102D sites suppress all recombination between the transgenes. It is also possible that the rates are determined entirely by local features and are not influenced by transgene sequences.

**Figure 4:**
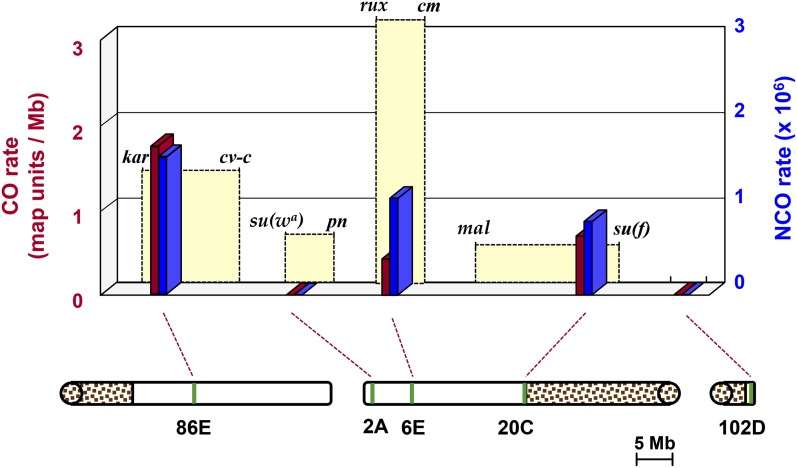
Recombination rates at various locations in the genome. Red bars are map units per megabase measured within *ry* at the endogenous location (86E) and at four different ectopic locations. Blue bars are the measured frequency of noncrossover gene conversion events at the same locations. Yellow segments are map units per megabase across a region flanking each site, according to Lindsley and Zimm (1992). The width of these bars is proportional to the size of the region that was used to calculate recombination rates. For 86E, this is 1.75 Mb (*kar* to *cv-c*); for 2A, it is 1.16 Mb (*su(w^a^)* to *pn*); for 6E, it is 1.6 Mb (*rux* to *cm*); for 20C, it is >2.2 Mb (*mal* to *su(f)*, which includes an unknown amount of unsequenced heterochromatin); and for 102D, it is 1.35 Mb (the entire assembled euchromatin on *4*). The drawings below represent *3R*, *X*, and *4R*. Stippled regions are pericentric heterochromatin. Green lines show approximate positions where recombination measurements were made.

It is unclear what local features affect recombination frequency. Comeron *et al.* (2012) noted that recombination is associated with transcribed regions. All four sites we assayed were selected by Bischof *et al.* (2007) as desirable locations for transgenesis because they are intergenic, so it seems unlikely that transcription influences our assay. Likewise, it seems unlikely that sequence motifs identified in fine-scale studies of crossover rates (Comeron *et al.* 2012; Singh *et al.* 2013) have an effect on this assay, since our insertion method resulted in more than four kb to the left of *ry^609^* and more than five kb to the right of *ry^606^* being identical between insertion locations. Nonetheless, it would interesting to incorporate motifs associated with elevated crossover rates into our transgenes to see whether an effect can be observed at any of the *X* chromosome sites we assayed.

Histone modifications are known to be important in specifying meiotic recombination initiation hotspots in yeast and mammals (Wu and Lichten 1994; Buard *et al.* 2009). It seems likely that chromatin modifications are likewise involved in the regulation of recombination in *Drosophila*, but initiation hotspots have not been identified and there have been no analyses of chromatin modifications in cells undergoing meiotic recombination.

In conclusion, we set out to develop an assay that could quantify the effects of gross position along a chromosome on the crossover/noncrossover decision. However, we were unable to detect any such effects, probably due to more localized effects on the ability to engage in recombination. A different transgene design, or possibly a gene other than *ry*, might mitigate these problems. One possibility is to surround the fragment with chromatin insulators (reviewed in Maeda and Karch 2007); however, it is unknown whether insulators function during meiosis or what aspects of recombination they might affect. An alternative is to increase the size of the transgenes. The genomic fragment we used includes parts of the genes immediately flanking *ry*, but it is possible that a larger genomic fragment will better recapitulate the endogenous properties of the region and resist any local effects of the insertion site. Current technologies allow integration of fragments exceeding 100 kb (*e.g.*, Venken *et al.* 2009), so it should be possible to develop a system that can assay the effects of chromosomal position on recombination frequency and outcome. Finally, it is possible that the insertion sites we selected are unusual in some way that masked the ability to detect effects of chromosomal position.

**Figure 5:**
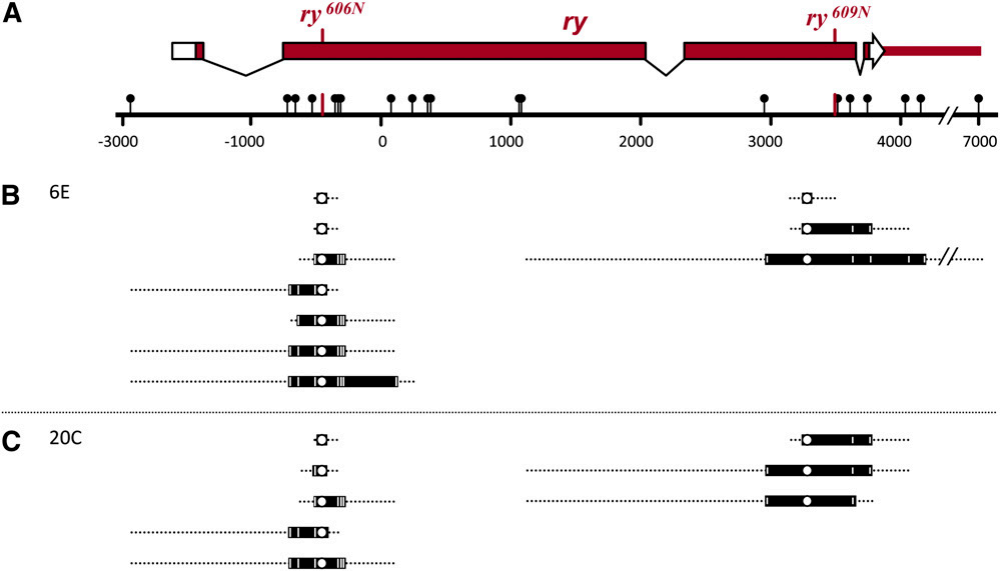
Gene conversion tracts at ectopic locations. (A) Schematic of the *rosy* locus. Intron/exon structure is shown, with coding sequences filled. The positions of the selected sites corresponding to the *ry^606N^* and *ry^609N^* mutations are shown. Heterologies between the *ry^606N^* and *ry^609N^* transgenes are indicated as lollipops on the scale bar. These are all single nucleotide polymorphisms except for the *Kpn*I site at −3149 and an insertion in *ry^609N^* at +4163 (Table 1). The scale is in bp, using the coordinate system of Coté *et al.* (Coté *et al.*). (B and C) Tract lengths observed in NCOs recovered at 6E (B) and 20C (C). Each bar represents an independent event, with the open circle denoting the selected marker (*ry^606N^* and *ry^609N^* mutant sites). Black bars represent the minimum tract length for each event, with co-converted sites marked by white lines. Dotted lines indicate the maximum tract length possible based on the next unconverted polymorphism.

## References

[bib1] Andersen S. L. Sekelsky J., 2010 Meiotic *vs.* mitotic recombination: two different routes for double-strand break repair: the different functions of meiotic *vs.* mitotic DSB repair are reflected in different pathway usage and different outcomes. Bioessays 32: 1058–1066 20967781 10.1002/bies.201000087 PMC3090628

[bib2] Aplin A. C. Kaufman T. C., 1997 Homeotic transformation of legs to mouthparts by proboscipedia expression in Drosophila imaginal discs. Mech. Dev. 62: 51–60 9106166 10.1016/s0925-4773(96)00649-1

[bib3] Barolo S. Carver L. A. Posakony J. W., 2000 GFP and beta-galactosidase transformation vectors for promoter/enhancer analysis in *Drosophila*. Biotechniques 29: 726–732 11056799 10.2144/00294bm10

[bib4] Beadle G. W., 1932 A possible influence of the spindle fibre on crossing-over in *Drosophila*. Proc. Natl. Acad. Sci. USA 18: 160–165 16577442 10.1073/pnas.18.2.160 PMC1076180

[bib5] Bhalla N. Dernburg A. F., 2008 Prelude to a division. Annu. Rev. Cell Dev. Biol. 24: 397–424 18597662 10.1146/annurev.cellbio.23.090506.123245 PMC4435778

[bib6] Bischof J. Maeda R. K. Hediger M. Karch F. Basler K., 2007 An optimized transgenesis system for Drosophila using germ-line−specific fC31 integrases. Proc. Natl. Acad. Sci. USA 104: 3312–3317 17360644 10.1073/pnas.0611511104 PMC1805588

[bib7] Blanton H. L. Radford S. J. McMahan S. Kearney H. M. Ibrahim J. G., 2005 REC, *Drosophila* MCM8, drives formation of meiotic crossovers. PLoS Genet. 1: 343–353 10.1371/journal.pgen.0010040 PMC1231718 16189551

[bib8] Buard J. Barthes P. Grey C. de Massy B., 2009 Distinct histone modifications define initiation and repair of meiotic recombination in the mouse. EMBO J. 28: 2616–2624 19644444 10.1038/emboj.2009.207 PMC2738703

[bib9] Chambers S. P. Prior S. E. Barstow D. A. Minton N. P., 1988 The pMTL nic- cloning vectors. I. Improved pUC polylinker regions to facilitate the use of sonicated DNA for nucleotide sequencing. Gene 68: 139–149 2851488 10.1016/0378-1119(88)90606-3

[bib10] Chovnick A. Gelbart W. M. McCarron M. Y. Pandey J., 1993 Studies on recombination in higher organisms, pp. 351–364 in Mechanisms in Recombination, edited by Grell R. F. Plenum Publishing Corporation, New York

[bib11] Cirulli E. T. Kliman R. M. Noor M. A., 2007 Fine-scale crossover rate heterogeneity in *Drosophila pseudoobscura*. J. Mol. Evol. 64: 129–135 17160365 10.1007/s00239-006-0142-7

[bib12] Comeron J. M. Ratnappan R. Bailin S., 2012 The many landscapes of recombination in *Drosophila melanogaster*. PLoS Genet. 8: e1002905 23071443 10.1371/journal.pgen.1002905 PMC3469467

[bib13] Coté B. Bender W. Curtis D. Chovnick A., 1986 Molecular mapping of the *rosy* locus in *Drosophila melanogaster*. Genetics 112: 769–783 2420682 10.1093/genetics/112.4.769 PMC1202776

[bib14] Dobzhansky T., 1930 Translocations involving the third and the fourth chromosomes of *Drosophila melanogaster*. Genetics 15: 347–399 17246603 10.1093/genetics/15.4.347 PMC1201068

[bib15] Finnerty V. G. Duck P. Chovnick A., 1970 Studies on genetic organization in higher organisms. II. Complementation and fine structure of the maroon-like locus of *Drosophila melanogaster*. Proc. Natl. Acad. Sci. USA 65: 939–946 5266164 10.1073/pnas.65.4.939 PMC283007

[bib16] Gelbart W. M. McCarron M. Y. Chovnick A., 1976 Extension of the limits of the XDH structural element in *Drosophila melanogaster*. Genetics 84: 211–232 826444 10.1093/genetics/84.2.211 PMC1213573

[bib17] Green M. M., 1955 Phenotypic variation and pseudo-allelism at the *forked* locus in *Drosophila melanogaster*. Proc. Natl. Acad. Sci. USA 41: 375–379 16589682 10.1073/pnas.41.6.375 PMC528098

[bib18] Hille R., 1996 The mononuclear molybdenum enzymes. Chem. Rev. 96: 2757–2816 11848841 10.1021/cr950061t

[bib19] Koehler K. E. Boulton C. L. Collins H. E. French R. L. Herman K. C., 1996 Spontaneous X chromosome MI and MII nondisjunction events in *Drosophila melanogaster* oocytes have different recombinational histories. Nat. Genet. 14: 406–414 8944020 10.1038/ng1296-406

[bib20] Lamb N. E. Freeman S. B. Savage-Austin A. Pettay D. Taft L., 1996 Susceptible chiasmate configurations of chromosome 21 predispose to non-disjunction in both maternal meiosis I and meiosis II. Nat. Genet. 14: 400–405 8944019 10.1038/ng1296-400

[bib21] Lindsley D. L. Zimm G. G., 1992 The Genome of Drosophila melanogaster. Academic Press, Inc., San Diego, CA

[bib22] Maeda R. K. Karch F., 2007 Making connections: boundaries and insulators in Drosophila. Curr. Opin. Genet. Dev. 17: 394–399 17904351 10.1016/j.gde.2007.08.002

[bib23] Martinez-Perez E. Colaiácovo M. P., 2009 Distribution of meiotic recombination events: talking to your neighbors. Curr. Opin. Genet. Dev. 19: 105–112 19328674 10.1016/j.gde.2009.02.005 PMC2729281

[bib24] Miller D. E. Takeo S. Nandanan K. Paulson A. Gogol M. M., 2012 A whole-chromosome analysis of meiotic recombination in *Drosophila melanogaster*. G3 (Bethesda) 2: 249–260 22384403 10.1534/g3.111.001396 PMC3284332

[bib25] Moens P. B. Kolas N. K. Tarsounas M. Marcon E. Cohen P. E., 2002 The time course and chromosomal localization of recombination-related proteins at meiosis in the mouse are compatible with models that can resolve the early DNA-DNA interactions without reciprocal recombination. J. Cell Sci. 115: 1611–1622 11950880 10.1242/jcs.115.8.1611

[bib26] Muller H. J., 1927 Artificial transmutation of the gene. Science 46: 84–87 17802387 10.1126/science.66.1699.84

[bib27] Otto S. P. Lenormand T., 2002 Resolving the paradox of sex and recombination. Nat. Rev. Genet. 3: 252–261 11967550 10.1038/nrg761

[bib28] Painter T. S. Muller H. J., 1929 Parallel cytology and genetics of induced translocations and deletions in Drosophila. J. Hered. 20: 287–298

[bib29] Phadnis N. Hyppa R. W. Smith G. R., 2011 New and old ways to control meiotic recombination. Trends Genet. 27: 411–421 21782271 10.1016/j.tig.2011.06.007 PMC3177014

[bib30] Plough H. H., 1917 The effect of temperature on linkage in the second chromosome of Drosophila. Proc. Natl. Acad. Sci. USA 3: 553–555 16586748 10.1073/pnas.3.9.553 PMC1091318

[bib31] Radford S. J. McMahan S. Blanton H. L. Sekelsky J., 2007 a Heteroduplex DNA in meiotic recombination in Drosophila *mei-9* mutants. Genetics 176: 63–72 17339219 10.1534/genetics.107.070557 PMC1893050

[bib32] Radford S. J. Sabourin M. M. McMahan S. Sekelsky J., 2007 b Meiotic recombination in Drosophila *Msh6* mutants yields discontinuous gene conversion tracts. Genetics 176: 53–62 17339220 10.1534/genetics.107.070367 PMC1893074

[bib33] Rørth P. Szabo K. Bailey A. Laverty T. Rehm J., 1998 Systematic gain-of-function genetics in Drosophila. Development 125: 1049–1057 9463351 10.1242/dev.125.6.1049

[bib34] Rubin G. M. Spradling A. C., 1982 Genetic transformation of *Drosophila* with transposable element vectors. Science 218: 348–353 6289436 10.1126/science.6289436

[bib35] Singh N. D. Aquadro C. F. Clark A. G., 2009 Estimation of fine-scale recombination intensity variation in the *white-echinus* interval of *D. melanogaster*. J. Mol. Evol. 69: 42–53 19504037 10.1007/s00239-009-9250-5 PMC2748731

[bib36] Singh N. D. Stone E. A. Aquadro C. F. Clark A. G., 2013 Fine-scale heterogeneity in crossover rate in the *garnet-scalloped* region of the *Drosophila melanogaster* X chromosome. Genetics 194: 375–387 23410829 10.1534/genetics.112.146746 PMC3664848

[bib37] Smith P. D. Finnerty V. G. Chovnick A., 1970 Gene conversion in *Drosophila*: Non-reciprocal events at the maroon-like cistron. Nature 228: 442–444 5482495 10.1038/228442a0

[bib38] Starz-Gaiano M. Cho N. K. Forbes A. Lehmann R., 2001 Spatially restricted activity of a Drosophila lipid phosphatase guides migrating germ cells. Development 128: 983–991 11222152 10.1242/dev.128.6.983

[bib39] Sturtevant A. H., 1913 The linear arrangement of six sex-linked factors in *Drosophila*, as shown by their mode of association. J. Exp. Biol. 14: 43–59

[bib40] Sturtevant A. H. Beadle G. W., 1936 The relations of inversions in the X chromosome of Drosophila melanogaster to crossing over and disjunction. Genetics 21: 554–604 17246812 10.1093/genetics/21.5.554 PMC1208722

[bib41] Venken K. J. Carlson J. W. Schulze K. L. Pan H. He Y., 2009 Versatile P[acman] BAC libraries for transgenesis studies in *Drosophila melanogaster*. Nat. Methods 6: 431–434 19465919 10.1038/nmeth.1331 PMC2784134

[bib42] Wu T. C. Lichten M., 1994 Meiosis-induced double-strand break sites determined by yeast chromatin structure. Science 263: 515–518 8290959 10.1126/science.8290959

[bib43] Youds J. L. Boulton S. J., 2011 The choice in meiosis - defining the factors that influence crossover or non-crossover formation. J. Cell Sci. 124: 501–513 21282472 10.1242/jcs.074427

